# Clinical and Ultrasonographic Features of Distal Ulnar Neuropathy: A Review

**DOI:** 10.3389/fneur.2019.00632

**Published:** 2019-06-24

**Authors:** Kristopher R. Karvelas, Francis O. Walker

**Affiliations:** ^1^Department of Orthopaedic Surgery and Rehabilitation, Wake Forest School of Medicine, Winston-Salem, NC, United States; ^2^Department of Neurology, Wake Forest School of Medicine, Winston-Salem, NC, United States

**Keywords:** ultrasound, sonography, ulnar nerve, guyon's canal, ulnar tunnel

## Abstract

Focal ulnar neuropathy at the wrist is a rare but problematic disorder often associated with the unique anatomy of this nerve as it courses through Guyon's canal, a superficial fibro-osseous tunnel in the proximal ulnar palm. The electrophysiologic features of this disorder have been well-characterized, but the sonographic anatomy of the nerve across the wrist and palm has yet to be systematically described in normal and abnormal states. In this review, we describe the basic anatomy and the sonographic appearance of the nerve in the wrist and palm in normals and individuals with pathology. The value of using US in conjunction with electrodiagnostic testing is emphasized as the two tests together provide critical information regarding etiology, predisposing factors, and functional significance. Furthermore, ultrasound is useful as a patient educational tool to promote behavioral changes that assist in nerve recovery when pathology is related to repetitive stress.

## Introduction

Ulnar mononeuropathy in the wrist and palm is uncommon but has a multitude of causes including trauma, occupational stressors, sports hazards, intrinsic, and extrinsic anatomical compression, and secondary effects of assistive devices. Although electrodiagnostic testing can often localize the injury distal to the forearm and assess electrophysiologic severity, it provides limited information regarding etiology. Imaging can enhance localization and, additionally, demonstrate the presence or absence of focal anatomic lesions along the course of the nerve. In a study of all ulnar neuropathies it was shown that in 74% of 281 cases, ultrasound (US) contributed to the evaluation in one of three categories: (1) uncovering a diagnosis not established by the electrodiagnostic testing, (2) enhancing the diagnosis by providing additional information regarding localization or predisposing anatomy, or (3) providing independent confirmation of the diagnosis (1).

Further support for the use of US for ulnar neuropathy at the wrist, comes from other studies showing US to be sensitive and specific in the workup of mononeuropathies in general. Specifically in ulnar mononeuropathy at the elbow it has been shown to have a sensitivity and specificity in the 74–76 and 72%, respectively (2, 3). In addition, these studies show that when using electrodiagnostic studies in conjunction with US, sensitivity is 93.3–98% (3). US has also been shown to be helpful in cases of electrically non-localizable ulnar mononeuropathy at the elbow (4). In carpal tunnel syndrome, a formal evidence based guideline has established the accuracy of sonographic diagnosis (Level A) (5). Relatively few cases of ulnar neuropathy at the wrist have been studied with US as the disorder is rare and sonographic evaluation of nerve a fairly recent development. However, it is now possible to non-invasively visualize the contents of Guyons canal and the small branches of the distal ulnar nerve which will be discussed in this review.

Ulnar mononeuropathies at the wrist/palm can be divided into acute and chronic etiologies. Acute injuries can be assessed with US to determine the location of nerve injury and if any structure (bone fragment, foreign body) is impinging the nerve (6). Penetrating trauma limited to the ulnar artery may also secondarily cause nerve injury from compression by the formation of a space occupying pseudoaneurysm. Chronic causes frequently include repetitive trauma from external compression. Internal causes for chronic compression include vascular etiologies and other space occupying forces. Table 1 provides an overview of some reported rare causes of distal ulnar neuropathy.

**Table 1:** Survey of reported etiologies of distal ulnar neuropathies.

**Acute etiologies**	**References**
Sports: cycling, fishing, kayaking, rowing, yoga, tug-of-war, weight lifting, push-ups	(7–13)
Iatrogenic causes during procedures: wrist arthrography, tendon transfers, carpal tunnel release	(7, 14, 15)
Toxins: Jellyfish sting	(16)
External compression: handcuffs	(17, 18)
**CHRONIC ETIOLOGIES**	
External compression: occupational, sports related, hobbies, assist device use by the disabled	(9, 19–25)
Vascular: hypothenar hammer syndrome, arterial thrombus, aneurysm, arteriovenous malformation	(26–28)
Non-vascular space occupying forces: accessory muscle impingement, thickened ligaments, osseous abnormalities, ganglions, lipomas, carpal tunnel syndrome	(29–37)
Structural irregularities due to systemic diseases: rheumatoid arthritis, polio	(7, 38)

US can play a critical role in investigating the causes for ulnar mononeuropathy in all the above scenarios. Repetitive trauma is thought to cause focal enlargement which can be localized on US (39). Vascular etiologies may be evaluated sonographically for structural irregularities, as well as with power Doppler assessing for normal flow. Space occupying lesions can obviously be identified as well-depending on the skill of the observer. US may be even more sensitive than MRI in this area, especially considering variability in MRI scanning resolution. However, this has not been directly investigated in this particular diagnosis. Paget et al. presents a case of a lipoma in Guyon's canal that was not appreciated on a 1.5T MRI but was later identified on surgical exploration (37). US evaluation was not reported in this case, but one would expect this to be visualized well on modern high frequency sonographic evaluation.

Given the relative scarcity of cases of ulnar neuropathy at the wrist and palm diagnosed and described with sonographic evaluation, the following discussion provides a description of normal anatomy of the ulnar nerve at the wrist and palm suitable to current instrumentation.

Sonographic anatomy of the ulnar nerve:

In order to perform a diagnostic sonographic evaluation, a comprehensive understanding of both neuroanatomy and surrounding musculoskeletal anatomy is needed. The ulnar nerve approaches the wrist by traveling through the ulnar forearm immediately deep to the flexor carpi ulnaris (FCU) and adjacent to the flexor digitorum profundas (FDP). The ulnar nerve gives off two branches as it approaches the wrist that do not travel through Guyon's canal. About 8.3 cm proximal, the dorsal ulnar cutaneous (DUC) nerve branches off and travels around the ulna to the dorsal hand (40). On sonographic evaluation by Kim et al. the palmar ulnar cutaneous (PUC) nerve was found to branch off the ulnar nerve at an average of 11.9 cm proximal to the pisiform, while the DUC was found to branch at an average of 7.1 cm proximal to the pisiform (41). The PUC travels along the ulnar aspect of the flexor tendons and the superficially over the transverse carpal ligament. The DUC travels laterally in an ulnar direction making its way to the dorsal hand between the metacarpals 4, 5.

When visualized on transverse view with US, the ulnar nerve at the wrist lies between the pisiform bone and ulnar artery at the proximal portion of the ulnar tunnel. The tunnel actually extends 2.5 mm proximal to the pisiform to the edge of the palmar carpal ligament. The actual size of the ulnar tunnel is variable as the boundaries of the tunnel change through its course, due to the surrounding anatomy (42, 43). In fact, the tunnel itself changes with wrist positioning (42). The transcarpal ligament lies above the carpal tunnel and then dives to form the proximal floor of the tunnel along with the flexor FDP. More distally, the floor is also formed by the pisohamate ligament, pisometacarpal ligament, and opponens digiti minimi (7, 42, 43). The proximal roof of the canal is composed of the palmar carpal ligament. The distal volar roof may also be composed of a portion of the palmaris brevis (43). The ulnar side of the canal is formed by the FCU, pisiform and ADM (42, 43). The radial side of the canal is composed of the digital flexor tendons, transverse carpal ligament and the hook of the hamate (7, 42).

The ulnar tunnel contains the ulnar nerve/branches, ulnar artery and variable communicating veins; all surrounded by fatty connective tissue (43). It has also been pointed out that anastomosing branches of the median nerve would also be located in the tunnel while traveling with the ulnar nerve (44). The nerve branches into the superficial and deep branches within Guyon's canal prior to the hook of the hamate. At the level of the hook of the hamate just after the deep branch starts to dive, the two branches are separated by the fibrous arch (pisohamate arch) of the flexor digiti minimi (FDM). More distally the muscle belly itself separates the two branches as it originates from the hamate and the aforementioned fibrous arch (7, 42, 43). The superficial branch of the ulnar nerve travels distally just after the bifurcation in Guyon's canal to lie directly over the hook of the Hamate and then travels through the ulnar palm. Just distal to the hamate the superficial ulnar nerve gives off the branch to digit 5. The superficial ulnar nerve and branches at this point lie between the palmaris brevis and FDM (43). Through its course it innervates one muscle (palmaris brevis) and then provides sensory innervation of the ulnar half of the fourth digit, fifth digit and ulnar hypothenar eminence (7, 45).

The deep branch first innervates the muscles of the hypothenar eminence (ADM, FDM, opponens digit minimi) (7, 45). It then dives around the ulnar slope of the hook of the Hamate and distally makes a sharp turn toward the radial palm as it travels to the thenar eminence. Prior to exiting Guyon's canal however, it initially travels through the hypothenar tunnel or pisohamate hiatus. The floor of this tunnel is comprised of the piso-hamate ligament and the roof by the tendinous origin of the hypothenar musculature (pisohamate arch) (7, 46). The branch that innervates the ADM may arise proximal or distal to the pisohamate arch. When it arises proximal it then travels distally over the pisohamate arch (7). It then travels under the FDM and opponens digit minimi before it courses dorsal to the flexor tendon group (7, 42). In the palm it innervates the interossei and third and fourth lumbricals before reaching the thenar eminence to innervate the adductor pollicis, flexor pollicis brevis, and finally the FDI (45).

## Accessory Muscles

Accessory muscles may be visualized at the ulnar palm. Dodds et al. found 22.4% incidence in 58 palms (47). Zeiss et al. found anomalous muscles in 25% of 36 palms examined (43). Harvie et al. (48) evaluated 116 asymptomatic wrists and 35% had an aberrant ADM. The most common is the accessory ADM which, when present, overlies the ulnar nerve and its branches in the canal (49). The characteristics of this muscle however may vary on sonographic evaluation depending on its origin which can be variable (50). On sonographic evaluation the muscle would thus typically be identified as a hypoechoic mass superficial to the ulnar nerve and lateral to the pisiform (39, 51, 52). Other possible accessory muscles include the reversed palmaris longus, high origin FDM, accessory palmaris longus, hypothenar adductor (7, 32). The palmaris brevis may be mistaken for an accessory muscle. This muscle can be identified by its location and fiber orientation. It originates from the transverse carpal ligament/palmar aponeurosis and inserts on the soft tissues of the ulnar palm in order to wrinkle the skin in this area.

## Definitions of Entrapment Zones

Initially, three zones of entrapment were described at the wrist to help localize lesions (53). Zone 1 is located proximal to or in Guyon's canal prior to the bifurcation of the superficial and deep branches. Both sensory and motor branches are located in this zone. Ulnar mononeuropathy located to Zone 1 causes deficits in all muscles and sensory areas innervated by the ulnar nerve in the hand. Zone 2 is distal to Zone 1. It contains only the deep branch of the ulnar nerve. Injury to the deep motor branch manifests in all innervated muscles of the ulnar hand with intact ulnar sensation. If compression occurs distally in this zone (pisohamate hiatus) distal to the take-off of the ADM then this muscle may be spared in a lesion of zone 2. Zone 3 is distal to zone 1 as well but is radial to zone 2, containing only the superficial branch of the ulnar nerve. A lesion in this zone manifests as ulnar sensory deficits in digits 5, ulnar half of 4 and the hypothenar eminence. The superficial ulnar branch innervated palmaris brevis is affected as well (7, 31, 45, 53). There have been updates and expansions to this description, however, the initial three zones described are most important to understand in terms of sonographic anatomy (54).

## Sonographic Examination of the Distal Ulnar Nerve

**Figure 1:**
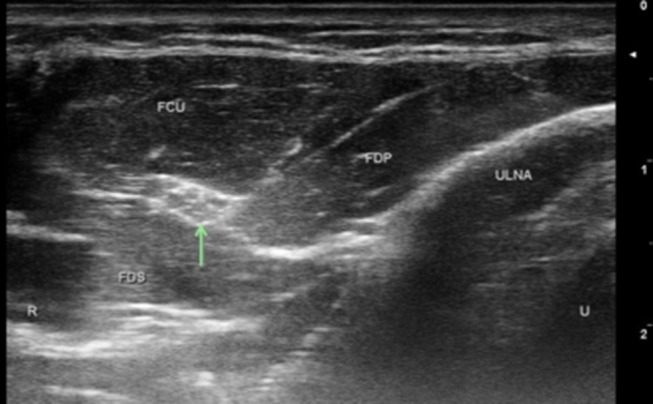
Normal ulnar nerve 5 cm below the elbow. Arrow, ulnar nerve; FDP, flexor digitorum profundas; FCU, flexor carpi ulnaris; FDS, flexor digitorum superficialis; U, ulnar; R, radial.

**Figure 2:**
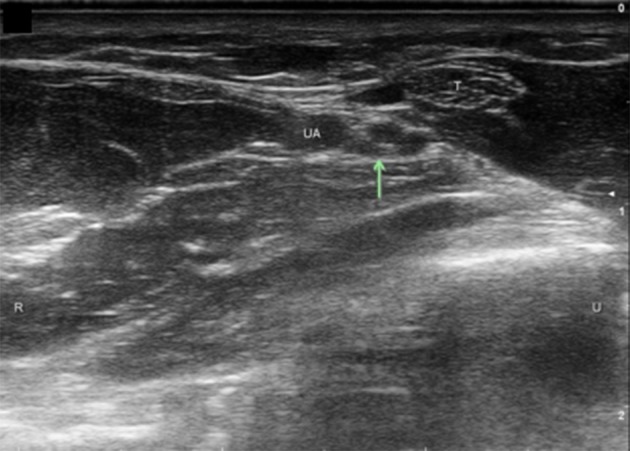
Ulnar nerve at level of FCU myotendinous junction. Arrow, ulnar nerve; T, tendon; UA, ulnar artery; U, ulnar; R, radial.

**Figure 3:**
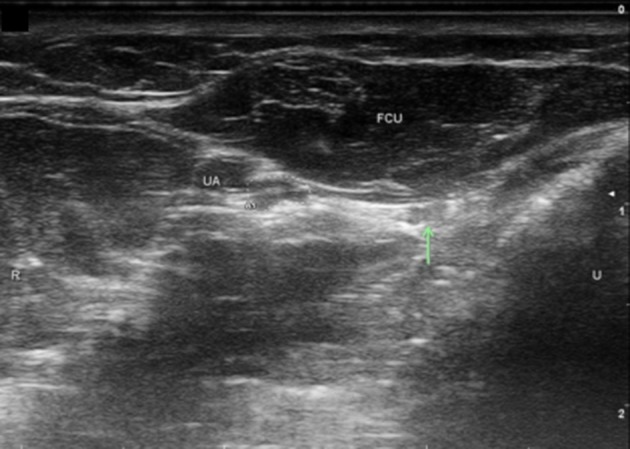
Dorsal ulnar cutaneous nerve (DUC) just distal to take-off. UA, ulnar artery; FCU, flexor carpi ulnaris; Arrow, DUC, Ulnar nerve circled; U, ulnar; R, radial.

**Figure 4:**
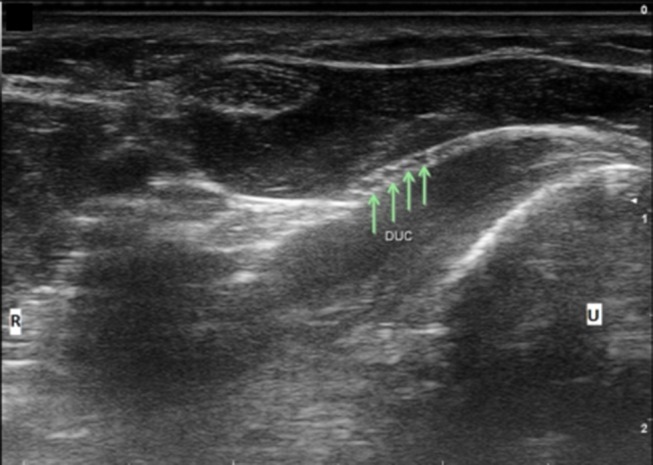
Dorsal ulnar cutaneous nerve (DUC) fascicles flattening as it courses under FCU. Arrows, DUC fascicles; U, ulnar; R, radial.

**Figure 5:**
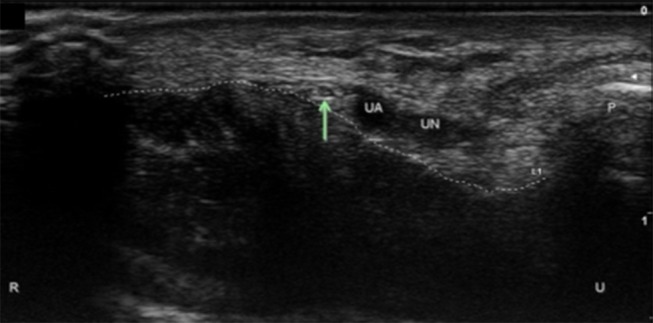
Palmar ulnar cutaneous nerve (PUC) at the wrist. Arrow, PUC; UA, ulnar artery; UN, ulnar nerve; P, pisiform; Dotted line, course of the transverse carpal ligament; U, ulnar; R, radial.

Ulnar nerve evaluation at the wrist starts with visualizing the nerve in the forearm and assessing the nerve as it approaches the wrist. It is easiest to identify the nerve in the proximal forearm as it rests between the FCU, FDP, and flexor digitorum superficialis in the space lateral to the ulna with the patient flexing their elbow to 90 degrees (Figure 1). It is also helpful to initiate evaluation at this point if one is considering in the differential ulnar nerve entrapment at the elbow and plans to image the ulnar nerve proximally. Follow the nerve distally through the forearm and notice its size and echogenic characteristics as it travels adjacent to the FCU. When at the level of the FCU myotendinous junction, make sure to differentiate the FCU tendon from the ulnar nerve as they are situated alongside each other (Figure 2). The DUC will be seen branching from the ulnar nerve (Figure 3). It will travel toward the ulna and as it courses under the FCU the fascicles will flatten as it makes its way to the dorsal wrist and then hand (Figure 4). The PUC may be seen branching more proximally in the forearm, though can be easier to identify superficial to the transverse carpal ligament at the palm (Figure 5). These two ulnar nerve sensory branches (1 cm distal to their branching origin) were found to average in size 0.3 mm^2^ for the PUC nerve and 1.5 mm^2^ for the DUC nerve (41).

**Figure 6:**
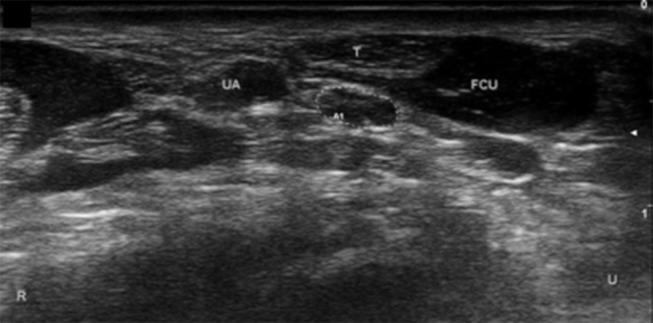
Ulnar nerve at distal wrist crease. UA, ulnar artery; FCU, flexor carpi ulnaris; T, FCU tendon. Ulnar nerve circled. U, ulnar; R, radial.

**Figure 7:**
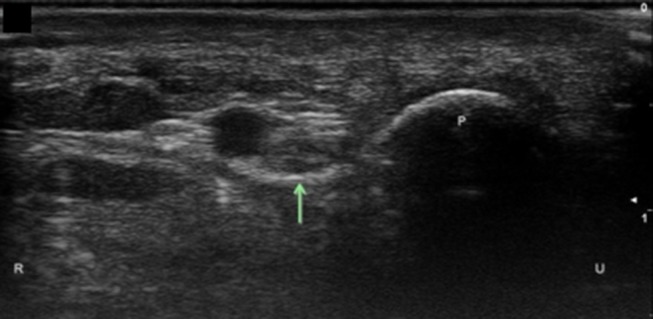
Ulnar nerve at the level of the pisiform. Arrow, ulnar nerve; P, pisiform; U, ulnar; R, radial.

**Figure 8:**
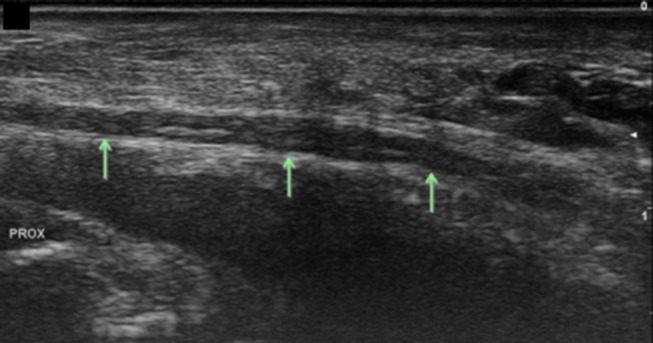
Ulnar nerve at the level of the pisiform, longitudinal view. Arrows, ulnar nerve; Prox, proximal.

At the distal wrist crease just prior to Guyon's canal the ulnar nerve can be measured (Figure 6). Cartwright et al. found the ulnar nerve size at the distal wrist crease in the dominant arm to be 5.9 mm^2^ in 60 subjects (55). Continue to follow the ulnar nerve to the wrist to the level of the pisiform. At the pisiform measure the ulnar nerve size (Figure 7). (56) found the ulnar nerve at Guyons canal to have a mean cross sectional area (CSA) of 3.1 ± 1 mm^2^ (56). Reckelhoff et al. evaluated the ulnar nerve and branches in Guyon's canal in 46 subjects/83 wrists (45). They found the mean CSA of the UN in Guyon's tunnel to be 6.0 ± 2.0 mm^2^ in men and 5.0 ± 1.0 mm^2^ in women. Also obtain longitudinal view of the nerve at this level (Figure 8).

**Figure 9:**
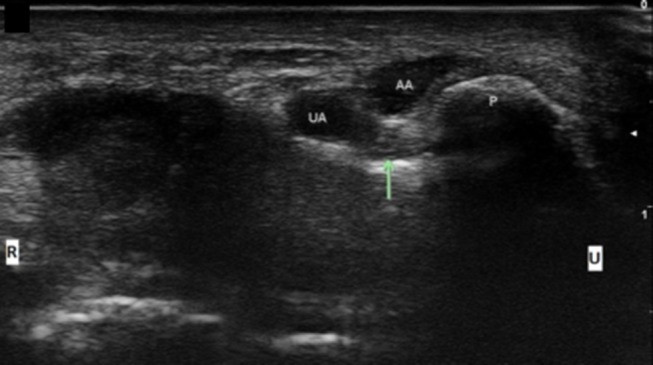
Ulnar nerve at the level of the pisiform with accessory adductor digiti minimi. UA, ulnar artery; AA, Accessory ADM; Arrow, ulnar nerve; P, pisiform; U, ulnar; R, radial.

**Figure 10:**
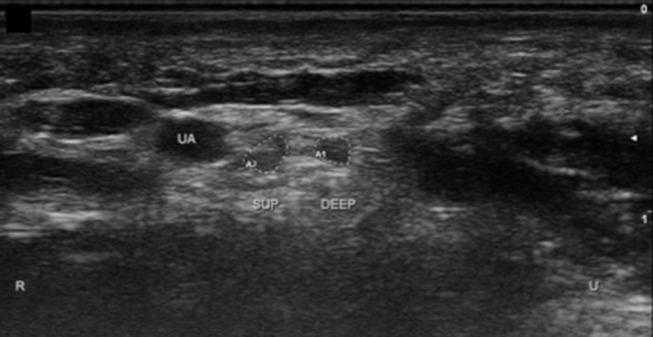
Ulnar nerve in Guyon's canal after bifurcation. UA, ulnar artery; Sup, superficial branch; Deep, deep branch; U, ulnar; R, radial.

**Figure 11:**
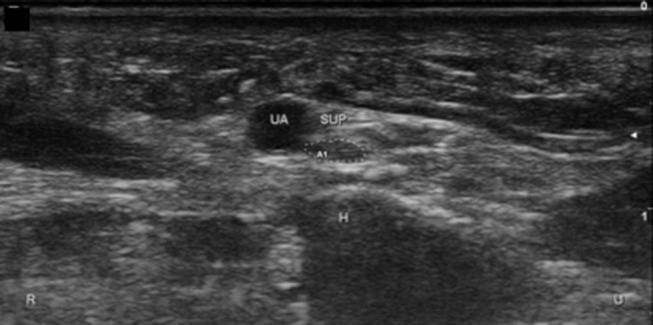
Superficial branch of the ulnar nerve at the hook of the hamate, short axis. UA, ulnar artery; Sup, superficial branch; H, hamate; U, ulnar; R, radial.

**Figure 12:**
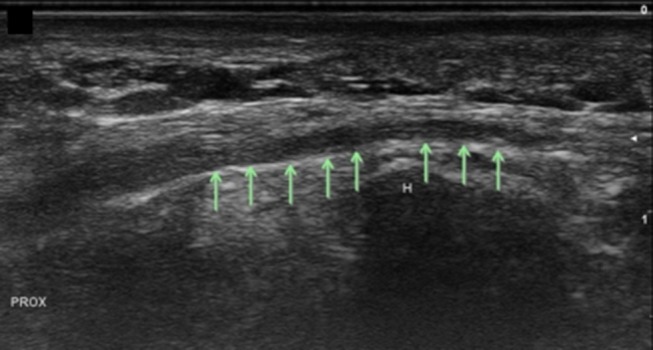
Superficial branch of the ulnar nerve at the hook of the hamate, long axis. Arrows, superficial ulnar branch; H, hamate; Prox, proximal.

**Figure 13:**
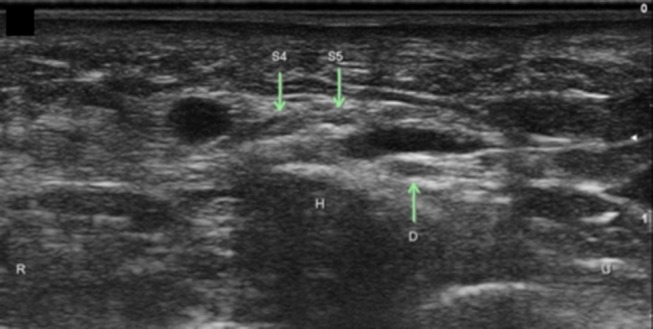
Superficial branch of the ulnar nerve with sensory branch to the ulnar 5th digit. H, hamate; S4, superficial branch to digit 4 and radial side of digit 5; S5, superficial branch to ulnar side of digit 5; D, Deep ulnar motor branch; R, radial; U, ulnar.

**Figure 14:**
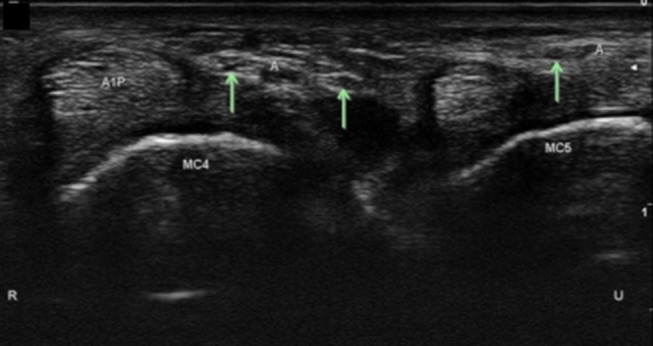
Digital sensory branches in the palm at level of the A1 pulley. A1P, A1 pulley; Arrows, distal sensory branches of the superficial branch of the ulnar nerve; A, artery; MC, metacarpal; R, radial; U, ulnar.

At the level of the pisiform, evaluate the ulnar artery for integrity and size, assessing for flow on Doppler. Also assess for accessory ADM superficial to the tunnel (Figure 9). Distal to the pisiform assess for any space occupying masses. Localize the bifurcation of the ulnar nerve into superficial and deep branches (Figure 10). Follow the superficial branch to the hook of the hamate in short axis (Figure 11). The superficial sensory branch has been found to average 3.9 mm^2^ CSA in one study (41) and 3.0 ± 1.0 mm^2^ in another (45). Rotate the probe to capture a longitudinal image of the superficial branch overlying the hook of the hamate and trace this distally evaluating for abnormality (Figure 12). Then trace distally in short axis assessing for the take-off of the sensory branch to the ulnar aspect of digit 5 at the level of the hamate (Figure 13). Follow these small sensory branches to the digits and notice the main trunk of the superficial sensory branch split mid-palm into the branches to digits 4 and the radial side of digit 5. In the distal palm at the level of the A1 pulley the three sensory branches can be well-visualized (Figure 14).

**Figure 15:**
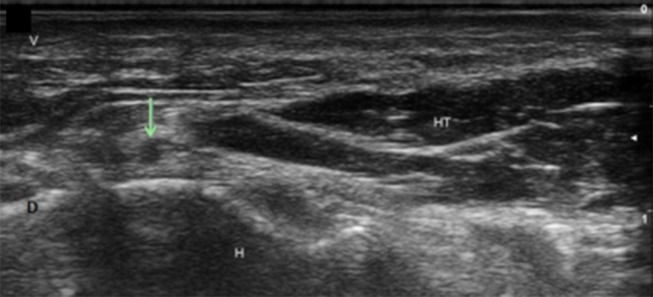
Deep branch of the ulnar nerve at the hamate. Arrow, deep branch; H, hamate; HT, hypothenar muscles; V, volar; D, dorsal.

**Figure 16:**
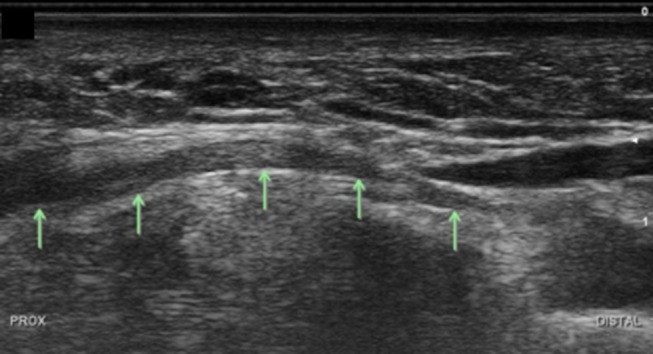
Deep branch of the ulnar nerve at level of the hamate, longitudinal view. Arrows, deep branch; Prox, proximal.

Return to the hook of the hamate and identify the deep motor branch. It may help to slide the transducer medially in order to assess the slope of the medial side of the hamate as the deep branch will begin to dive here (Figure 15). In this view, the deep branch is intimately superficial to the medial hook of the hamate. The CSA of the motor branch was 2.0 ± 1.0 mm^2^ in men and women. At the slope of the hamate turn the probe to capture a longitudinal view of the nerve and to appreciate its approach to course deep in the palm (Figure 16). At this point identify the muscles that surround the nerve. Medially will be the ADM and superficially the FDM.

It is always helpful and recommended to assess the asymptomatic side, despite having literature that describes the average cross sectional areas at specific landmarks as noted above. Side to side variability has been found to be low when assessing the distal ulnar nerve, allowing for the asymptomatic side to serve as a control (55, 56).

## Conclusion

As the use of high frequency US becomes more common in neuromuscular medicine our understanding of neuromuscular sono-anatomy improves as well. This has already been shown to improve our ability to confirm diagnosis of mononeuropathies as well as establish the diagnosis when electrodiagnostic workup is inconclusive. US also helps uncover the etiology of the injury as well as to correlate predisposing anatomical factors (intrinsic and extrinsic) with actual pathophysiology. Recent studies have suggested that US echointensity ratios in distally innervated muscles may help assess relative axon loss (57). If further research validates this approach, it may constitute another non-invasive way to enhance diagnosis of distal ulnar neuropathy. A final value of US, is that it helps establish an anatomic baseline for future comparison, even if normal (58). It is our opinion that neuromuscular US should be used in the workup of every case or suspected case of ulnar mononeuropathy at the wrist due to the ease of use, current quality of imaging and low cost of application.

## Author Contributions

KK: Image acquisition, drafting of the manuscript and critical revision of the manuscript for important intellectual content. FW: Image acquisition and critical revision of the manuscript for important intellectual content.

### Conflict of Interest Statement

The authors declare that the research was conducted in the absence of any commercial or financial relationships that could be construed as a potential conflict of interest.

## Abbreviations

US: ultrasound
FDI: first dorsal interosseous
ADM: Adductor digiti minimi
FCU: flexor carpi ulnaris
FDP: flexor digitorum profundas
DUC: dorsal ulnar cutaneous
PUC: palmar ulnar cutaneous
FDM: flexor digiti minimi
CSA: cross sectional area.

## References

[1] Simon P Contribution of ultrasonography to the evaluation of peripheral nerve disorders. Clin Neurophysiolgy. (2018) 48:119–23 10.1016/j.neucli.2018.01.001 29415823

[2] van Veen K Wesstein M van Kasteel V. Ultrasonography and electrodiagnostic studies in ulnar neuropathy: an examination of the sensitivity and specificity and the correlations between both diagnostic tools. J Clin Neurophysiol. (2015) 32:240–3. 10.1097/WNP.0000000000000148 25394273

[3] Beekman R Visser L Verhagen W. Ultrasonography in ulnar neuropathy at the elbow: a critical review. Muscle Nerve. (2011) 43:627–35. 10.1002/mus.22019 21484821

[4] Alrajeh M Preston D. Neuromuscular ultrasound in electrically non-localizable ulnar neuropathy. Muscle Nerve. (2018) 58:655–9. 10.1002/mus.26291 29981241

[5] Cartwright M Hobson-Webb L Boon A Alter K Hunt C Flores V. Evidence-based guideline: neuromuscular ultrasound for the diagnosis of carpal tunnel syndrome. Muscle Nerve. (2012) 46:287–93. 10.1002/mus.23389 22806381

[6] Padua L Di Pasquale A Liotta G Granata G Pazzaglia C Erra C. (2013). Ultrasound as a useful tool in the diagnosis and management of traumatic nerve lesions. Clin Neurophysiol. 124:1237–43. 10.1016/j.clinph.2012.10.024 23380690

[7] Chen S Tsai T. Ulnar tunnel syndrome. J Hand Surg Am. (2014) 39:571–9. 10.1016/j.jhsa.2013.08.102 24559635

[8] Akuthota V Plastaras C Lindberg K Tobey J Press J Garvan C. The effect of long-distance bicycling on ulnar and median nerves: an electrophysiologic evaluation of cyclist palsy. Am J Sports Med. (2005) 33:1224–30. 10.1177/0363546505275131 16000656

[9] Horner L Edelsohn L Gakhal M. Yoga induced acute ulnar nerve compression by a ganglion cyst in Guyon's canal. Del Med J. (2013) 85:369–73. 24654357

[10] Muppidi S Orengo J Lutz A. Overexertion-related focal ulnar neuropathy. Muscle Nerve. (2016) 53:989–91. 10.1002/mus.25011 26663314

[11] Patterson J Jaggars M Boyer M. Ulnar and median nerve palsy in long-distance cyclists. A prospective study. Am J Sports Med. (2003) 31:585–9. 10.1177/03635465030310041801 12860549

[12] Seguti V Júnior A Flores L Ferreira L. Acute lesion of the motor branch of the ulnar nerve in the wrist after tug-of-war training. Rev Bras Ortop. (2011) 46:741–4. 10.1016/S2255-4971(15)30336-0 27047837 PMC4799355

[13] Walker F Troost B. Push-up palmar palsy. JAMA. (1988) 1;259:45–6. 10.1001/jama.1988.03720010025026 3334767

[14] Giuffre J Shin A Bilateral ulnar neuropathy secondary to volar displacement of flexor tendons following open carpal tunnel release: case report. J Hand Surg. (2011) 36A:2027–9. 10.1016/j.jhsa.2011.09.005 22018476

[15] McCleave M. Ulnar nerve injury after flexor tendon grafting. J Hand Surg Asian Pac Vol. (2016) 21:425–7. 10.1142/S2424835516720218 27595967

[16] Peel N Kandler R. Localized neuropathy following jellyfish sting. Postgrad Med J. (1990) 66:953–4. 10.1136/pgmj.66.781.953 1980012 PMC2429735

[17] Grant AC Cook AA. A prospective study of handcuff neuropathies. Muscle Nerve. (2000) 23:933–8. 10.1002/(SICI)1097-4598(200006)23:6<933::AID-MUS14>3.0.CO;2-G 10842271

[18] Satkunam L Zochodne D. Bilateral ulnar handcuff neuropathies with segmental conduction block. Muscle Nerve. (1995) 18:1021–3. 10.1002/mus.880180916 7643865

[19] Capitani D Beer S. Handlebar palsy—a compression syndrome of the deep terminal (motor) branch of the ulnar nerve in biking. J Neurol. (2002) 249:1441–5. 10.1007/s00415-002-0864-4 12382163

[20] Dozono K Hachisuka A Wada F Hachisuka K. Peripheral neuropathies in nonparetic upper extremities of stroke patients induced by excessive use of a walking device. J Stroke Cerebrovasc Dis. (2015) 24:1841–7. 10.1016/j.jstrokecerebrovasdis.2015.04.022 25997977

[21] Ginanneschi F Filippou G Milani P Biasella A Rossi A. Ulnar nerve compression neuropathy at Guyon's canal caused by crutch walking: case report with ultrasonographic nerve imaging. Arch Phys Med Rehabil. (2009) 90:522–4. 10.1016/j.apmr.2008.09.568 19254622

[22] Hankey G Gubbay S. Compressive mononeuropathy of the deep palmar branch of the ulnar nerve in cyclists. J Neurol Neurosurg Psychiatry. (1988) 51:1588–90. 10.1136/jnnp.51.12.1588 2851643 PMC1032781

[23] Pyo J Pasquina P DeMarco M Wallach R Teodorski E Cooper R. Upper limb nerve entrapment syndromes in veterans with lower limb amputations. PM R. (2010) 2:14–22. 10.1016/j.pmrj.2009.10.002 20129508

[24] Shaffer SW Alexander K Huffman D Kambe C Miller R Miller JH. Median and ulnar neuropathies in US army dental personnel at Fort Sam, Houston, Texas. US Army Med Dep J. (2014) 65–73. Available online at: http://search.ebscohost.com/login.aspx?direct=true&db=mth&AN=95582277&site=ehost-live (accessed June 6, 2019). 24706246

[25] Streib E Sun S. Distal ulnar neuropathy in meat packers. An occupational disease? J Occup Med. (1984) 26:842–3. 10.1097/00043764-198411000-00015 6502288

[26] Monacelli G Rizzo M Spagnoli A Monarca C Scuderi N. Ulnar artery thrombosis and nerve entrapment at guyon's canal: our diagnostic and therapeutic algorithm. In Vivo. (2010) 24:779–82. 20952749

[27] Ozdemir O Calisaneller T Altinors N. Compression of the ulnar nerve in Guyon's canal by an arteriovenous malformation. J Hand Surg Eur. (2007) 32:600–1. 10.1016/J.JHSE.2007.04.005 17950236

[28] Yoshii S Ikeda K Murakami H Gazzeri G Humansky F Ulnar nerve compression secondary to ulnar artery true aneurysm at Guyon's canal. J Neurosurg Sci. (1999) 434:295–7. 10864392

[29] Chen W Barnwell J Li Y Smith B Li Z. An ulnar intraneural ganglion arising from the pisotriquetral joint: case report. J Hand Surg Am. (2011) 36:65–7. 10.1016/j.jhsa.2010.08.033 21093175

[30] Coraci D Luchetti R Paolasso I Santilli V Padua L. Intermittent ulnar nerve compression due to accessory abductor digiti minimi muscle: crucial diagnostic role of nerve ultrasound. Muscle Nerve. (2015) 52:463–4. 10.1002/mus.24660 25808715

[31] Erkin G Uysal H Kele I Aybay C Özel S. Acute ulnar neuropathy at the wrist: acase report and review of the literature. Rheumatol Int. (2006) 27:191–6. 10.1007/s00296-006-0166-8 16896989

[32] Failla J. The hypothenar adductor muscle: an anomalous intrinsic muscle compressing the ulnar nerve. J Hand Surg Am. (1996) 21:366–8. 10.1016/S0363-5023(96)80345-5 8724462

[33] Gozke E Dortcan N Kocer A Cetinkaya M Akyuz G Us O. Ulnar nerve entrapment at wrist associated with carpal tunnel syndrome. Neurophysiol Clin. (2003) 33:219–22. 10.1016/j.neucli.2003.08.002 14672822

[34] Inaparthy P Anwar F Botchu R Jèahnich H Katchburian M. Compression of the deep branch of the ulnar nerve in Guyon's canal by a ganglion: two cases. ArchOrthop Trauma Surg. (2008) 128:641–3. 10.1007/s00402-008-0636-4 18509691

[35] Kang S Yang S Yoon J Kang H Won S. Effect of carpal tunnel syndrome on the ulnar nerve at the wrist. sonographic and electrophysiologic studies. J Ultrasound Med. (2016) 35:37–42. 10.7863/ultra.15.02064 26589645

[36] Lal R Raj S. Guyons canal syndrome due to accessory palmaris longus muscle: aetiological classification: a case report. Cases J. (2009) 2:9146. 10.1186/1757-1626-2-9146 20062663 PMC2803943

[37] Paget J Patel N Manushakian J Ulnar nerve compression in Guyon's canal: MRI does not always have the answer. J Surg Case Rep. (2013) 2013:rjs043 10.1093/jscr/rjs043 24963936 PMC3579532

[38] Tsai H Hung T Chen C Lieu F Cho H Tung T Chen S. Prevalence and risk factors for upper extremity entrapment neuropathies in polio survivors. J Rehabil Med. (2009) 41:26–31. 10.2340/16501977-0290 19197565

[39] Cartwright M Walker F. Neuromuscular ultrasound in common entrapment neuropathies. Muscle Nerve. (2013) 48:696–704. 10.1002/mus.23900 23681885

[40] Goto A Kunihiro O Murase T Moritomo H. The dorsal cutaneous branch of the ulnar nerve: an anatomical study. Hand Surg. (2010) 15:165–8. 10.1142/S021881041000493X 21089189

[41] Kim K Lee S Park B Kim D. Sonoanatomy of the sensory branches of the ulnar nerve below the elbow in healthy subjects. Muscle Nerve. (2018) 57:569–73. 10.1002/mus.25959 28877548

[42] Ombaba J Kuo M Rayan G. Anatomy of the ulnar tunnel and the influence of wrist motion on its morphology. J Hand Surg Am. (2010) 35:760–8. 10.1016/j.jhsa.2010.02.028 20438994

[43] Zeiss J Jakab E Khimji T Imbriglia J. The ulnar tunnel at the wrist (Guyon's canal): normal MR anatomy and variants. Am J Roentgenol. (1992) 158:1081–5. 10.2214/ajr.158.5.1566671 1566671

[44] Depukat P Mizia E Klosinski M Dzikowska M Klimek-Piotrowska W Mazur M. Anatomy of Guyon's canal—a systematic review. Folia Med Cracov. (2014) 54:81–6. 25648313

[45] Reckelhoff K Li J Kaeser M Haun D Kettner N Ultrasound evaluation of the normal ulnar nerve in guyon's tunnel: cross-sectional area and anthropometric measurements. J Med Ultrasound. (2015) 23:171–6. 10.1016/j.jmu.2015.09.002

[46] Francisco B Agarwal J. Giant cell tumor of tendon sheath in Guyon's canal causing ulnar tunnel syndrome. A case report and review of the literature. Eplasty. (2009) 9:e8. 19252681 PMC2637121

[47] Dodds G Hale D Jackson W Incidence of an anatomic variance in Guyon's canal. J Hand Surg. (1990) 15A:352–5. 10.1016/0363-5023(90)90122-8 2324469

[48] Harvie P Patel N Ostlere S. Prevalence and epidemiological variation of anomalous muscles at Guyon's canal. J Hand Surg Br. (2004) 29:26–9. 10.1016/j.jhsb.2003.08.004 14734065

[49] Bozkurt M Tagil S Ozçakar L Ersoy M Tekdemir I. Anatomical variations as potential risk factors for ulnar tunnel syndrome: a cadaveric study. Clin Anat. (2005) 18:274–80. 10.1002/ca.20107 15832354

[50] Claassen H Schmitt O Schulze M Wree A. Variation in the hypothenar muscles and its impact on ulnar tunnel syndrome. Surg Radiol Anat. (2013) 35:893–9. 10.1007/s00276-013-1113-5 23558800

[51] Jacobson JA Fundamentals of Musculoskeletal Ultrasound. Second Edition. Philadelphia, PA: Saunders/Elsevier, (2013).

[52] Timins M. Muscular anatomic variants of the wrist and hand: findings on MR imaging. AJR Am J Roentgenol. (1999) 172:1397–401. 10.2214/ajr.172.5.10227524 10227524

[53] Gross M Gelberman R The anatomy of the distal ulnar tunnel. Clin Orthop Relat Res. (1985) 196:238–47. 10.1097/00003086-198506000-00033 3995823

[54] Coraci D Loreti1 C Piccinini G Doneddu P Biscotti S Padua L. Ulnar neuropathy at wrist: entrapment at a very congested site. Neurol Sci. (2018) 39:1325–31. 10.1007/s10072-018-3446-7 29779137

[55] Cartwright M Shin H Passmore L Walker F. Ultrasonographic findings of the normal ulnar nerve in adults. Arch Phys Med Rehabil. (2007) 88:394–6. 10.1016/j.apmr.2006.12.020 17321837

[56] Tagliafico A Martinoli C. Reliability of side-to-side sonographic cross-sectional area measurements of upper extremity nerves in healthy volunteers. J Ultrasound Med. (2013) 32:457–62. 10.7863/jum.2013.32.3.457 23443186

[57] Kim J Seok H Kim B. The significance of muscle echo intensity on ultrasound for focal neuropathy: the median- to ulnar-innervated muscle echo intensity ratio in carpal tunnel syndrome. Clin Neurophysiol. (2016) 127:880–5. 10.1016/j.clinph.2015.04.055 25998202

[58] Walker F Cartwright M Alter K Visser Leo. Hobson-Webb L Padua L. Indications for neuromuscular ultrasound: expert opinion and review of the literature. Clin Neurophysiol. (2018) 129:2658–79 10.1016/j.clinph.2018.09.013 30309740

